# Knowledge, attitude, and perception of Pakistani populations toward monkeypox: a cross-section study

**DOI:** 10.3389/fcimb.2024.1449096

**Published:** 2025-02-04

**Authors:** Humayun Yousaf, Abdul Qadeer, Muhammad Sohail, Maqbool Khan, Muhammad Farooq, Zakir Khan, Dalia Fouad, Yu-Chia Liu, Chien-Chin Chen

**Affiliations:** ^1^ Department of Medical Laboratory Technology, Kohat University of Science and Technology, Kohat, Pakistan; ^2^ Department of Cell Biology, School of Life Sciences, Central South University, Changsha, China; ^3^ Key Laboratory of Imaging Diagnosis and Minimally Invasive Intervention Research, Zhejiang Engineering Research Center of Interventional Medicine Engineering and Biotechnology, Key Laboratory of Precision Medicine of Lishui City, Lishui Hospital of Zhejiang University, Lishui, China; ^4^ School of Computing Sciences, Pak-Austria Fachhochschule: Institute of Applied Sciences and Technology, Haripur, KPK, Pakistan; ^5^ Department of Microbiology University of Swabi, Swabi, Pakistan; ^6^ School of Pharmacy and Biomolecular Sciences, Royal College of Surgeons (RCSI) University of Medicine and Health Sciences, Dublin, Ireland; ^7^ Department of Zoology, College of Science, King Saud University, Riyadh, Saudi Arabia; ^8^ Department of Stomatology, Ditmanson Medical Foundation Chia Yi Christian Hospital, Chiayi, Taiwan; ^9^ Department of Pathology, Ditmanson Medical Foundation Chia-Yi Christian Hospital, Chiayi, Taiwan; ^10^ Department of Cosmetic Science, Chia Nan University of Pharmacy and Science, Tainan, Taiwan; ^11^ Doctoral Program in Translational Medicine, National Chung Hsing University, Taichung, Taiwan; ^12^ Department of Biotechnology and Bioindustry Sciences, College of Bioscience and Biotechnology, National Cheng Kung University, Tainan, Taiwan

**Keywords:** monkeypox, endemic, knowledge, attitude, perception, Pakistan

## Abstract

**Objective:**

The reappearance of monkeypox in non-endemic countries has preceded critical public health concerns. The public’s adherence to preventative measures is influenced by their understanding of the infectious monkeypox virus (Mpox), attitude toward it, and perceptions of it. We conducted this study to evaluate the general population’s knowledge, attitude, and perceptions of Mpox in Pakistan.

**Methods:**

From August 15 to August 30, 2022, 3465 participants in this cross-sectional study provided information via an online survey. The survey has 37 questions divided into four categories: sociodemographic, knowledge, attitude, and perceptions of Mpox. Statistical analyses were performed in Jupyter Notebook using Python 3 and the Pandas, Matplotlib, and stats libraries.

**Results:**

The chi-square test and regression analysis evaluated factors related to Mpox knowledge, attitude, and perception of three thousand four hundred sixty-five participants. Among the participants, about 79.51% (2755) were male, and 32.99% (1143) had post-graduation level education. About 521(99.24) participants with post-graduation education have positive knowledge with a p-value of 0.0001. Approximately 66.78% (2314) of participants answered that Mpox is prevalent in African countries. Almost 85.69% (2969) stated that a virus causes Mpox, and more than 72.18% (2501) said that Mpox spreads through contaminated surfaces. About 60.52% (2097) of participants answered that antivirals are required in treating Mpox patients. Around more than half of the participants, 52.64% (1824), believe that those who receive the chickenpox vaccination are protected against Mpox. It’s interesting to note that most research participants felt favorably about Mpox. Around 90.22% (3126) of participants believed Mpox would be effectively eradicated. Most participants, 86.7% (3004), believe that people should care more about one another nowadays, and 81.36% (2819) believe visiting areas with a Mpox outbreak is risky. About 83.95% (2909) answered that during interaction with the Mpox patient, they’d dress in the appropriate personal protective clothing, like masks, gloves, and gowns. At the same time, 77.66% (2691) responded that they would perform hand hygiene after touching the patients’ surroundings, like beds, tables, doors, etc.

**Conclusions:**

Maximum research participants showed good attitudes and knowledge about Mpox. However, it is essential to start and execute a planned planning framework for public health awareness to avoid the occurrence and spread of Mpox in Pakistan.

## 1 Introduction

Mpox, formerly known as monkeypox, is a zoonotic viral infection that has gained international attention due to its rising incidence and potential to spread globally. Initially identified in 1958, Mpox is primarily found in tropical rainforest regions of Central and West Africa, with occasional outbreaks outside these areas due to international travel and global interconnectedness (Di Giulio and Eckburg, 2004; Kaler et al., 2022; Shafaati et al., 2022; Khattak et al., 2023a; Ali et al., 2024). The recent surge in cases worldwide has raised concerns about the disease’s potential to become a significant public health threat, particularly in regions where it has not been documented, such as South Asia (Bunge et al., 2022). Mpox is similar to the smallpox virus, sharing many clinical features, including fever, lymphadenopathy, and a characteristic rash. However, Mpox has distinct transmission modes, primarily spreading through close contact with infected individuals, contaminated materials, or animal vectors (Anwar et al., 2023; Khattak et al., 2023a; Khattak et al., 2023b; Zandi et al., 2023). While Mpox is generally less severe than smallpox, the absence of widespread immunity due to the cessation of smallpox vaccination programs increases susceptibility among younger populations. With no specific treatment currently available, prevention through awareness and behavioral modification remains critical to managing the spread of Mpox (Reynolds et al., 2006; Anwar et al., 2023).

The recent outbreak started because of the West African clade (Ogoina et al., 2019) when the United Kingdom Health Security Agency recorded the first case of Mpox on May 7, 2022. Since then, a steady stream of Mpox cases has been reported to the World Health Organization (WHO) (Sah et al., 2022a; Zumla et al., 2022). On July 23, 2022, WHO classified Mpox as a public health emergency of worldwide concern (W.H.O, 2022). As of 1^st^ December 2022, there were more than 81,000 Mpox cases worldwide, an increase from the previous year (W.H.O.a.o.D.A).

Despite Mpox being recorded in several Southeast Asian nations (India, Thailand, and Indonesia) (World Health Organization; REUTERS, 2022), there has been no confirmed Mpox in Pakistan. The National Institute of Health debunked the rumor of Mpox cases in Pakistan on May 24, 2022, despite reports of two uncommon instances of the zoonotic Mpox (c.N.D.n.R.o.M.A.D, 2022). The lack of awareness among the general population of the WHO is one obstacle to preventing the re-emergence of Mpox (World Health Organization, 2022), with most people, doctors, and politicians in impoverished nations unaware of Mpox (EMA, 2022). As a preventive measure, it is crucial to spread “knowledge” to limit the rapid spread of the disease. The scientific and media communities should be engaged to drive the “attitude” of every section of the community. While the world is already facing one pandemic, the goal is to contain the disease with appropriate “practices” based on the “Knowledge, Attitudes and Perceptions” regarding this infectious disease. A community’s collective knowledge, attitudes, and perceptions significantly determine how far a virus may spread within a given area (Ahmed et al., 2021; Tahir et al., 2021; Khattak et al., 2022a). There is a lack of knowledge about fundamental health rights because of ignorance and illiteracy. General populations should be better equipped to alert the public if a pandemic occurs. Targeting this general population is done mainly for this purpose.

Another significant difficulty is the health system’s reactivity, exacerbated by the fact that we typically uncover issues after they have become complicated and take a reactive rather than proactive approach. If Mpox spreads, an already creaking healthcare system will be at the point of disaster. There is no viral diagnostic facility in Pakistan. Still, the health department has proclaimed that in an emergency, samples may be sent outside for testing, which increases the possibility of the virus spreading. To deal with this circumstance, it is necessary to understand the disease’s presenting signs and symptoms to guarantee the prompt quarantine of suspected patients rather than symptomatic therapies. Hospitals should also have well-equipped isolation facilities ready to place patients under quarantine immediately to stop the spread of the dangerous virus.

This study aims to assess the knowledge, attitudes, and perceptions of the Pakistani population regarding Mpox to inform public health strategies. By identifying current knowledge levels and misconceptions, the study seeks to provide insights to guide the development of targeted educational campaigns, improve community engagement, and enhance preventive measures against Mpox in Pakistan. Understanding these factors is crucial to ensuring that public health interventions are effectively designed and implemented, ultimately contributing to the broader goal of safeguarding public health in the face of emerging infectious diseases.

## 2 Materials and Methods

### 2.1 Study design

From August 15 to August 30, 2022, we performed cross-sectional web-based research with the general Pakistani population. According to the most recent census, Pakistan’s population is 207.07 million (Pakistan., B.o.S.G.o, 2017). The study was open to all Pakistanis aged 18 and older who could understand English. The required sample was determined using the Raosoft sample size calculator. The estimated minimum sample size needed was 385. A 50% response distribution was considered for the sample estimate, along with a 5% margin of error and a 95% confidence level. To get more exact and accurate results, we gathered 3465 respondents for the survey.

### 2.2 Data collection tool

To collect data, we conducted an online survey. Cronbach’s Alpha test was utilized to evaluate the reliability of the knowledge attitude and perceptions questions independently. The value of Cronbach’s Alpha for knowledge is 0.81, and attitude is 0.77 and 0.69 for perception. It shows that Knowledge questions are more strongly consistent than Attitude and Perception. Recruitment was conducted using convenience sampling combined with a simplified snowball sampling technique. The survey was configured to accept only one response per participant, verified through their email addresses. Participants could complete and submit the survey using a computer or a mobile device. The questionnaire was divided into four sections: Data on sociodemographic characteristics were included in the first part, in addition to gender, age, Marital status, Residence, Administrative Units, education, and employment status. The second section contained twenty-three multiple-choice questions designed to measure knowledge. The third had nine attitude-testing questions, while the fourth contained six developed perception-testing questions on the Mpox. The study’s instruments were modified from previous studies (Harapan et al., 2017; Harapan et al., 2018; Harapan et al., 2019; Harapan et al., 2020; Khattak et al., 2021; Alshahrani et al., 2022; Khattak et al., 2022a; Khattak et al., 2022b). “Yes,” “No,” and “Not sure” were the only options given to respondents for each question. The survey was written in English.

### 2.3 Data collection procedure

No personal information was required in the survey, like a person’s name, address, or any other information that may be used to identify them. Using a specific platform Google Forms link, participants were invited to complete a structured questionnaire (see **Supplementary Materials 1**). We circulated it through social media sites like Twitter, Facebook, and WhatsApp. The survey link was shared with different groups on WhatsApp and Facebook, and the admin and members of these groups were requested to share the link to get enough responses. Before starting the replies, each respondent was asked to confirm they had informed permission by clicking the consent declaration. “I do with this, after reading the aims of the study, engage in the survey supplying my information by answering questions rationally and willingly,” was the informed consent statement offered to the respondents. Respondents filled out the survey and clicked the “submit” button to send it to our platform for data collection. To confirm the authenticity of the responses, all questions were compulsory to be answered.

### 2.4 Study variables

A total of 23 knowledge questions with “Yes,” “No,” and “Not Sure” response options were used to gauge how well-informed the Pakistani people were about Mpox. The knowledge score was 0 (lowest) to 23 (highest). Age, which was divided into groups of 18 to 30 years, 31 to 49 years, and more or equal ≥50 years; gender (male or female); marital status (single or married); participants’ residency area of Pakistan (Khyber Pakhtunkhwa, Sindh, Azad Kashmir, Islamabad Capital Territory, Punjab, Baluchistan, and Gilgit-Baltistan); and urban or rural area. Furthermore, participants were asked about employment status, and education was divided into five levels: postgraduate, graduate, college, high school, read and write,/primary school. Six questions focused on perception, and nine on attitude and practice in anticipating the disease.

### 2.5 Statistical analysis

The responses collected via Google Forms were exported to Microsoft Excel. Statistical analysis was conducted in Jupyter Notebook using Python 3 and the Pandas, Matplotlib, and stats libraries. Factors linked with Mpox knowledge, attitude, and perception were discovered using chi-square and Multiple regression analysis. Descriptive statistics such as frequency and percentage were employed to demonstrate the demographic features of the research participants. The chi-square test and Multiple regression analysis were conducted to evaluate response variables and explanatory factors. A P-value of 0.05 was set to determine statistical significance.

### 2.6 Ethical consideration

The methods utilized in this investigation follow the Helsinki Declaration and the ethical guidelines of relevant national and institutional committees on human experimentation. The Ethical Research Committee of the Department of Microbiology approved the study at the University of Swabi, Pakistan (F.No.1(3)-Micro-8/UOS/2022/1878).

## 3 Result

### 3.1 Characteristics of respondents

In the final analysis, 3465 research participants were recruited, with 79.51% being men, just 20.49% being females, and 44.53% being Khyber Pakhtunkhwa citizens. Most respondents were 18–30 (43.29%) and from Urban areas (69.7%). 32.99% of participants have a postgraduate level of education. According to the marital status findings, most participants (54.55%) were single, and 27.47% worked full-time. **Table 1** provides a summary of the research participants’ demographic characteristics.

**Table 1:** Demographic characteristics of participants(N=3465).

S.No.	Variable	Unique Values	Frequency	Percentage (%)
1	Gender	Male	2755	79.51
		Female	710	20.49
2	Age	18-30 years	1500	43.29
		31-49 years	1350	38.96
		≥50 years	615	17.75
3	Marital status	Single	1890	54.55
		Married	1575	45.45
4	Residence	Urban	2415	69.7
		Rural	1050	30.3
5	Administrative Units	Khyber Pakhtunkhwa	1543	44.53
		Sindh	525	15.15
		Azad Kashmir	418	12.06
		Islamabad Capital Territory	317	9.15
		Punjab	285	8.23
		Baluchistan	210	6.06
		Gilgit-Baltistan	167	4.82
6	Education	Postgraduate	1143	32.99
		Graduate	945	27.27
		Intermediate/College	758	21.88
		High School	525	15.15
		Read and write/Primary School	94	2.71
7	Employment Status	Full-Time	952	27.47
		Part-Time	805	23.23
		Unemployed	740	21.36
		Worker	520	15.01
		Retired	273	7.88
		Housewife	175	5.05

### 3.2 Knowledge

**Tables 2**, **3**; **Supplementary Material S2** display the replies to inquiries testing participants’ knowledge of Mpox. Of the male participants, 79.58% (565) have high positive knowledge about Mpox compared to female participants. The 18-29 participants have more positive knowledge than others, with a p-value of 0.0001. Same in marital status, 78.94% (1492) married participants have high positive knowledge related to single participants with a p-value of 0.0008. Approximately 48.43% (1678) affirmed that there had not been any reports of Mpox in humans in Pakistan. Nevertheless, several queries received incorrect responses, such as, A human Mpox case has been confirmed in Pakistan, according to around 27.19% (942) of respondents. Almost 85.69% (2969) stated that a virus causes Mpox, and about 72.18% (2501) said that Mpox spreads through contaminated surfaces. At the same time, approximately 77.14% (2673) of the participants believed that Mpox is easily transmitted from human to human, 48.46% (1679) by Bit of an infected Monkey, and 41.96% (1454) by airborne transmission. The majority of participants thought the section on measures to stop disease transmission was that proper handwashing 80.12% (2776), adequate social distance 82.08% (2844), avoiding handshakes and hugs 83.69% (2900), and cleaning and disinfecting surfaces 74.69% (2588) were utterly essential. Furthermore, 66.46% (2303) of participants said that quarantining sick people alone was a preventative process to stop the spread of disease.

**Table 2:** Participant’s knowledge of Mpox (N= 3465).

S. No.	Questions/variable	Values	Frequency	Percentage
1	Is monkeypox (Mpox) prevalent in African countries?	Yes	2314	66.78
		No	630	18.18
		Not Sure	521	15.04
2	There are many cases of Mpox in Pakistan.	No	1678	48.43
		Yes	942	27.19
		Not Sure	845	24.39
3	Is Mpox a viral disease infection?	Yes	2969	85.69
		Not Sure	392	11.31
		No	104	3
4	Does Mpox spread through contaminated surfaces?	Yes	2501	72.18
		Not Sure	569	16.42
		No	395	11.4
5	Is Mpox easily transmitted from human to human?	Yes	2673	77.14
		No	528	15.24
		Not Sure	264	7.62
6	Does Mpox spread by airborne transmission?	Yes	1454	41.96
		No	1046	30.19
		Not Sure	965	27.85
7	Does Mpox spread by a Bit of an infected Monkey?	Yes	1679	48.46
		No	1243	35.87
		Not Sure	543	15.67
8	Measures to prevent the spread of the Mpox disease include [Proper hand washing]	Yes	2776	80.12
		Not Sure	360	10.39
		No	329	9.49
9	Measures to prevent the spread of the Mpox disease include [adequate social distance from a symptomatic individual]	Yes	2844	82.08
		Not Sure	418	12.06
		No	203	5.86
10	Measures to prevent the spread of the disease include [Avoiding handshakes and hugs]	Yes	2900	83.69
		Not Sure	448	12.93
		No	117	3.38
11	Measures to prevent the spread of the disease include [Clean and disinfecting surfaces]	Yes	2588	74.69
		Not Sure	553	15.96
		No	324	9.35
12	Measures to prevent the spread of the disease include [Self-quarantine or isolation if sick]	Yes	2303	66.46
		Not Sure	637	18.38
		No	525	15.15
13	Common symptoms include [Headache]	Yes	2730	78.79
		No	525	15.15
		Not Sure	210	6.06
14	Common symptoms include [Back pain]	Yes	2205	63.64
		Not Sure	1050	30.3
		No	210	6.06
15	Common symptoms include [fever]	Yes	2939	84.82
		Not Sure	418	12.06
		No	108	3.12
16	Common symptoms include [Lymphadenopathy (swollen lymph nodes)]	Yes	2206	63.67
		Not Sure	1044	30.13
		No	215	6.2
17	Do Mpox, smallpox, and chickenpox have similar signs and symptoms?	Yes	1797	51.86
		Not Sure	1453	41.93
		No	215	6.2
18	Rashes on the skin are one of the signs or symptoms of human Mpox	Yes	2311	66.7
		Not Sure	840	24.24
		No	314	9.06
19	Papules on the skin are one of the signs or symptoms of human Mpox	Yes	2425	69.99
		Not Sure	832	24.01
		No	208	6
20	Vesicles on the skin are one of the signs or symptoms of human Mpox	Yes	2516	72.61
		Not Sure	531	15.32
		No	418	12.06
21	Are antivirals required in the treatment of human Mpox patients?	Yes	2097	60.52
		Not Sure	746	21.53
		No	622	17.95
22	Are people who get the chickenpox/smallpox vaccines immunized against Mpox?	Yes	1824	52.64
		Not Sure	1029	29.7
		No	612	17.66
23	Is there a specific treatment for Mpox?	Yes	1611	46.49
		Not Sure	1133	32.7
		No	721	20.81

**Table 3:** Association of knowledge with demographic variables of participants(N=3465).

S.No.	Variable	Unique values	Good knowledge (%)	Poor knowledge (%)	Chi-square	Significance (p-value)
1	Gender	Male	565 (79.58)	145 (20.42)	4.0310	0.0447
		Female	2094 (76.01)	661 (23.99)		
2	Age	18-30 years	1244 (82.93)	256 (17.07)	56.9896	0.0001
		31-49 years	969 (71.78)	381 (28.22)		
		≥50 years	446 (72.52)	169 (27.48)		
3	Marital status	Single	1167 (74.1)	408 (25.9)	11.3047	0.0008
		Married	1492 (78.94)	398 (21.06)		
4	Residence	Urban	841 (80.1)	209 (19.9)	9.5078	0.0020
		Rural	1818 (75.28)	597 (24.72)		
5	Administrative Units	Khyber Pakhtunkhwa	207 (65.3)	110 (34.7)	508.1123	0.0001
		Sindh	157 (94.01)	10 (5.99)		
		Azad Kashmir	271 (95.09)	14 (4.91)		
		Islamabad Capital Territory	206 (98.1)	4 (1.9)		
		Punjab	1184 (76.73)	359 (23.27)		
		Baluchistan	393 (94.02)	25 (5.98)		
		Gilgit-Baltistan	241 (45.9)	284 (54.1)		
6	Education	Postgraduate	521 (99.24)	4 (0.76)	449.7543	0.0001
		Graduate	838 (88.68)	107 (11.32)		
		Intermediate/College	779 (68.15)	364 (31.85)		
		High School	494 (65.17)	264 (34.83)		
		Read and write/Primary School	27 (28.72)	67 (71.28)		
7	Employment Status	Full-Time	750 (78.78)	202 (21.22)	339.5244	0.0001
		Part-Time	172 (98.29)	3 (1.71)		
		Unemployed	698 (86.71)	107 (13.29)		
		Worker	328 (63.08)	192 (36.92)		
		Retired	442 (59.73)	298 (40.27)		
		Housewife	269 (98.53)	4 (1.47)		

### 3.3 Attitudes

**Tables 4**, **5**; **Supplementary Material S2** depict the participants’ attitudes toward Mpox. Remarkably, most research participants had a positive attitude toward Mpox. Most participants, 90.22% (3126), believed Mpox could be managed effectively. Around 86.7% (3004) of participants said that individuals should show more concern for one another nowadays. Furthermore, responding that they would go above and beyond to take care of themselves and their family members was 87.04% (3016) of the participants. There are now sufficient preventative and control methods for Mpox, according to about 72.44% (2510) of respondents. Around approximately 69.96% (2424) of participants have bad feelings toward Mpox; considering the possibility that it may spread globally, 69.7% (2415) think Mpox can be transmitted to Pakistan, and 81.36% (2819) believe that visiting the region where the Mpox pandemic is prevalent is risky. Also, 83.67% (2899) of participants think Mpox might burden the impacted nations’ medical systems. While 82.94% (2874) of participants are concerned with learning more about Mpox, **Tables 4**, **5** provide the details of these sections.

**Table 4:** Attitudes towards Mpox (N=3465).

S. No.	Questions/variable	Values	Frequency	Percentage
1	Attitudes [Do you agree that Mpox will finally be successfully controlled]?	Yes	3126	90.22
		Not Sure	317	9.15
		No	22	0.63
2	Attitudes [People must take more care of each other now]	Yes	3004	86.7
		Not Sure	240	6.93
		No	221	6.38
3	Attitudes [I will do everything I can to protect myself and my family]	Yes	3016	87.04
		Not Sure	228	6.58
		No	221	6.38
4	I think that there are currently enough prevention and control measures for Mpox	Yes	2510	72.44
		No	512	14.78
		Not Sure	443	12.78
5	I have bad feelings toward the Mpox that it might become a worldwide pandemic	Yes	2424	69.96
		No	600	17.32
		Not Sure	441	12.73
6	I think Mpox can be transmitted to Pakistan	Yes	2415	69.7
		Not Sure	833	24.04
		No	217	6.26
7	I think that it is dangerous to travel to the country’s epidemic with Mpox	Yes	2819	81.36
		No	407	11.75
		Not Sure	239	6.9
8	I think that Mpox can add a new burden on the healthcare system of the affected countries	Yes	2899	83.67
		Not Sure	443	12.78
		No	123	3.55
9	I am interested in learning more about Mpox	Yes	2874	82.94
		Not Sure	347	10.01
		No	244	7.04

**Table 5:** Association of attitudes with demographic variables of participants(N=3465).

S.No.	Variable	Unique values	Good knowledge (%)	Poor knowledge (%)	Chi-square	Significance (p-value)
1	Gender	Male	690 (97.18)	20 (2.82)	18.9652	0.0001
		Female	2554 (92.7)	201 (7.3)		
2	Age	18-30 years	1397 (93.13)	103 (6.87)	9.8940	0.0071
		31-49 years	1254 (92.89)	96 (7.11)		
		≥50 years	593 (96.42)	22 (3.58)		
3	Marital status	Single	1469 (93.27)	106 (6.73)	0.5995	0.4388
		Married	1775 (93.92)	115 (6.08)		
4	Residence	Urban	940 (89.52)	110 (10.48)	42.3720	0.0001
		Rural	2304 (95.4)	111 (4.6)		
5	Administrative Units	Khyber Pakhtunkhwa	306 (96.53)	11 (3.47)	70.3183	0.0001
		Sindh	160 (95.81)	7 (4.19)		
		Azad Kashmir	270 (94.74)	15 (5.26)		
		Islamabad Capital Territory	208 (99.05)	2 (0.95)		
		Punjab	1391 (90.15)	152 (9.85)		
		Baluchistan	391 (93.54)	27 (6.46)		
		Gilgit-Baltistan	518 (98.67)	7 (1.33)		
6	Education	Postgraduate	518 (98.67)	7 (1.33)	143.9408	0.0001
		Graduate	938 (99.26)	7 (0.74)		
		Intermediate/College	1017 (88.98)	126 (11.02)		
		High School	677 (89.31)	81 (10.69)		
		Read and write/Primary School	94 (100.0)	0 (0.0)		
7	Employment Status	Full-Time	853 (89.6)	99 (10.4)	100.5648	0.0001
		Part-Time	175 (100.0)	0 (0.0)		
		Unemployed	795 (98.76)	10 (1.24)		
		Worker	479 (92.12)	41 (7.88)		
		Retired	671 (90.68)	69 (9.32)		
		Housewife	271 (99.27)	2 (0.73)		

### 3.4 Perception

**Tables 6**, **7**; **Supplementary Material S2** include the results of the perception questionnaire evaluating participants’ perceptions of several characteristics of Mpox. In total, 79.48% (2754) stated “handling and eating adequately cooked meat might help avoid disease, 80.49% (2789) said that medical professionals should be informed of sick patients’ recent travel history, and 76.8% (2661) participants discussed Mpox prevention with family and friends. About 83.95% (2909) answered that during interaction with the Mpox patient, they would wear the proper personal protective clothing, like masks, gloves, and gowns. At the same time, 77.66% (2691) of the participants answered that they would wash their hands after contacting the patients’ surroundings, such as beds, tables, doors, etc. However, 83.52% (2894) of participants said they would avoid inappropriate close contact and practice social distancing from patients and other healthcare professionals.

**Table 6:** Perception towards Mpox (N=3465).

S. No.	Questions/variable	Values	Frequency	Percentage
1	Perceptions toward Mpox [During the outbreak, eating well-cooked and safely handled meat can prevent Mpox]	Yes	2754	79.48
		No	359	10.36
		Not Sure	352	10.16
2	Perceptions toward Mpox [Sick patients should share their recent travel history with health care providers]	Yes	2789	80.49
		No	538	15.53
		Not Sure	138	3.98
3	Perceptions toward Mpox [I discussed Mpox prevention with my family and friends].	Yes	2661	76.8
		No	638	18.41
		Not Sure	166	4.79
4	During interaction with the Mpox patient, I will wear personal protective equipment such as masks, gloves, gowns, etc.	Yes	2909	83.95
		Not Sure	323	9.32
		No	233	6.72
5	I will perform hand hygiene after touching the patients’ beds, tables, doors, etc.	Yes	2691	77.66
		Not Sure	454	13.1
		No	320	9.24
6	I will avoid unnecessary close contact and practice social distancing from patients and other healthcare workers	Yes	2894	83.52
		No	324	9.35
		Not Sure	247	7.13

**Table 7:** Association of perception with demographic variables of participants(N=3465).

S.No.	Variable	Unique values	Good knowledge (%)	Poor knowledge (%)	Chi-square	Significance (p-value)
1	Gender	Male	600 (84.51)	110 (15.49)	96.4008	0.0001
		Female	2620 (95.1)	135 (4.9)		
2	Age	18-30 years	1388 (92.53)	112 (7.47)	216.1709	0.0001
		31-49 years	1336 (98.96)	14 (1.04)		
		≥50 years	496 (80.65)	119 (19.35)		
3	Marital status	Single	1452 (92.19)	123 (7.81)	2.3987	0.1214
		Married	1768 (93.54)	122 (6.46)		
4	Residence	Urban	939 (89.43)	111 (10.57)	28.0980	0.0001
		Rural	2281 (94.45)	134 (5.55)		
5	Administrative Units	Khyber Pakhtunkhwa	304 (95.9)	13 (4.1)	84.3134	0.0001
		Sindh	150 (89.82)	17 (10.18)		
		Azad Kashmir	271 (95.09)	14 (4.91)		
		Islamabad Capital Territory	210 (100.0)	0 (0.0)		
		Punjab	1376 (89.18)	167 (10.82)		
		Baluchistan	391 (93.54)	27 (6.46)		
		Gilgit-Baltistan	518 (98.67)	7 (1.33)		
6	Education	Postgraduate	520 (99.05)	5 (0.95)	182.3439	0.0001
		Graduate	932 (98.62)	13 (1.38)		
		Intermediate/College	973 (85.13)	170 (14.87)		
		High School	706 (93.14)	52 (6.86)		
		Read and write/Primary School	89 (94.68)	5 (5.32)		
7	Employment Status	Full-Time	944 (99.16)	8 (0.84)	715.1354	0.0001
		Part-Time	79 (45.14)	96 (54.86)		
		Unemployed	790 (98.14)	15 (1.86)		
		Worker	481 (92.5)	39 (7.5)		
		Retired	663 (89.59)	77 (10.41)		
		Housewife	263 (96.34)	10 (3.66)		

## 4 Discussion

As global cases of Mpox continue to rise, Pakistan needs to enhance its preparedness. While public health officials focus on prevention and epidemic management, success depends heavily on public engagement. Understanding the Pakistani population’s knowledge, attitudes, and perceptions towards Mpox is essential for developing effective public health interventions, including educational campaigns and community participation in management, prevention, and care efforts.

This study reveals a high awareness of Mpox among the Pakistani public, with 85.69% of respondents correctly identifying it as a viral infection. These findings highlight a notable difference compared to previous studies that have reported significant knowledge gaps between healthcare workers and the public regarding other infectious diseases, such as measles and dengue (Ahmed et al., 2022; Alsanafi et al., 2022; Nath et al., 2022; Sallam et al., 2022). This lack of information is unsurprising given that Mpox is a reemerging infectious disease with no documented cases in Pakistan or South Asia. Our results are consistent with findings from a Malaysian study that reported over 95% of respondents were aware of dengue fever during an outbreak despite limited knowledge about its transmission and treatment (Naing et al., 2011). Similarly, a pilot study among hospital visitors in Karachi, Pakistan, found that only 38.5% of participants had adequate knowledge of viral diseases, even though 90% knew their existence (Itrat et al., 2008). Another study from Pakistan noted that only 35% of individuals, regardless of socioeconomic status, had sufficient awareness of dengue during the epidemic, paralleling data on public awareness of the COVID-19 pandemic (Syed et al., 2010).

This consistency in public awareness of infectious disease symptoms and preventive measures reflects an encouraging baseline for future public health campaigns targeting emerging threats like Mpox. For instance, studies on COVID-19 indicated that 90-96% of Pakistani participants were knowledgeable about its symptoms, transmission routes, and prevention (Khattak et al., 2022b). However, further research has shown that approximately 90% of individuals were familiar with various infectious diseases’ clinical manifestations and symptoms (Khattak et al., 2021). This underscores the importance of enhancing public understanding of Mpox, especially as an emerging viral threat (Cheema et al., 2017). The relative absence of Mpox awareness in Pakistan is partly due to the country’s climate and the historical lack of outbreaks, as Mpox is primarily endemic to tropical rainforest regions (Damon, 2011; Durski et al., 2018). For travelers to endemic areas, such as the Democratic Republic of Congo (DRC) and China, raising awareness about Mpox and related diseases is essential for encouraging preventive measures (Nolen et al., 2016; Hadi et al., 2020).

Our study found that participants were well informed about the causes of Mpox, with notable awareness of its transmission through contaminated surfaces (72.18%), human-to-human contact (77.14%), airborne exposure (41.96%), and animal bites (48.46%). Key preventive measures recognized by participants included proper hand washing (80.12%), social distancing (82.08%), avoiding physical contact (83.69%), disinfecting surfaces (74.69%), and self-quarantine if sick (66.46%). The most well-known symptoms included fever (84.82%), headache (78.79%), rash (66.7%), and vesicles on the skin (72.61%). These findings are consistent with previous studies that reported high public knowledge of disease prevention and common symptoms (Jamil et al., 2022). Given the similarities between smallpox and Mpox regarding symptoms, transmission, and prevention strategies, the widespread recognition of Mpox symptoms may be partially attributed to public familiarity with smallpox (Petersen and Damon, 2015; Bunge et al., 2022; Kmiec and Kirchhoff, 2022). However, only 52.64% of participants knew that the smallpox vaccine also offers protection against Mpox, suggesting the need for more education on this aspect of prevention (Earl et al., 2004; Edghill-Smith et al., 2005).

Most participants displayed positive attitudes towards prevention, agreeing that isolating infected individuals and adopting protective measures, such as face masks and hand hygiene, could help control the spread. This is likely influenced by recent experiences during the COVID-19 pandemic, when preventive measures were the primary defense before vaccines were available (Hemati et al., 2022). Moreover, the guidance from global health authorities like the CDC and WHO may have contributed to the public’s adherence to recommended practices.

Gender and education levels were significant factors influencing knowledge and perceptions. Similar to earlier research, our study found that male participants were more aware of Mpox (Nath et al., 2022). This may be linked to the higher prevalence of Mpox cases among men who have sex with men, as noted in recent outbreaks (Seang et al., 2022; Bragazzi et al., 2023). A pooled meta-analysis showed that over 91% of recent Mpox cases involved sexual contact (Sah et al., 2022b), and a UK health center reported that all detected Mpox cases were among men who have sex with men (Girometti et al., 2022). Participants with higher education levels demonstrated better knowledge than those with only primary or secondary education, consistent with previous findings that associate higher education with greater health awareness and protective behaviors (Li et al., 2020; Raghupathi and Raghupathi, 2020). This may be attributed to increased access to information through the media, the internet, and educational resources.

Geographic and socio-economic disparities also played a role, with urban residents showing greater awareness than those in rural areas, reflecting findings from similar studies in other developing nations (Wen et al., 2020). This discrepancy can be attributed to greater access to mainstream media, the Internet, and educational resources in urban settings (Ahdab, 2020). Limited resources for health education in rural areas and insufficient support for medical professionals may contribute to lower awareness levels in these communities.

Mpox, as an emerging infectious disease, poses a significant threat to global public health (Kmiec and Kirchhoff, 2022; Shafaati and Zandi, 2022). Preventive measures are crucial in mitigating the spread of Mpox, especially without a dedicated vaccine. Raising awareness through targeted campaigns and educational initiatives can prevent outbreaks of contagious diseases like Mpox, COVID-19, influenza, and dengue from escalating into pandemics (Ismail and Ahmed, 2010; Tahir et al., 2020; Mahmood et al., 2022). Public engagement is critical; without widespread cooperation, efforts to enhance public health interventions through studies evaluating knowledge and attitudes are less effective (Tomar et al., 2021; Riccò et al., 2022).

Given Pakistan’s diverse population and varying access to information, knowledge levels about Mpox are likely to differ. Those without internet access or living in areas with limited outreach may have lower awareness, emphasizing the need for targeted education programs. Our study highlights the importance of multifaceted interventions, such as community engagement and media campaigns, which have been successful in the past and can be adapted to improve Mpox awareness and prevention (Ahinkorah et al., 2020; Geoffrion et al., 2020; Ali et al., 2022).

Healthcare professionals, social workers, and community partners should cooperate with multi-sectoral organizations to gather resources for Mpox awareness. Combining situational data into local and national studies can guide efficient practices and update policy. A national data dashboard for real-time decision-making would further support public health works. Taking the initiative measures, informed via lessons from COVID-19 and other epidemics, is essential for strengthening Pakistan’s response approaches.

## 5 Study limitations and strengths

Numerous limitations should be considered when assessing the outcomes of this research. Firstly, the use of a convenience sampling method leads to the potential for selection bias. Secondly, the survey was circulated online through various social media platforms, which could lead to bias, as individuals from lower socioeconomic backgrounds might have been excluded, limiting the capacity to generalize the results to the larger population. Thirdly, since the research relied on previously published studies in English, there could be a language bias in the data, which may have influenced participants’ responses, especially in relation to complex or technical topics. Respondents may have also given socially desirable answers to questions about their attitudes and perceptions, aligning their responses with what they believed was expected of them. Fourth, the survey is subject to recall bias, which could have affected the accuracy of the participants’ responses.

Additionally, since most participants were married and residing in urban areas, any results stratified by these factors should be taken with concern. Also, the sample included 79.51% males, which may affect the generalizability of the outcomes, remarkably in relation to gender-specific trends, and should be admitted as a limitation. Another issue is that the questionnaire was only available in English, which could have excluded less educated individuals, specifically in a developing country, concerning the representativeness of the sample. Yet, the large sample size of the study strengthens its outcomes. Eventually, the insights from this research could be effective for health officials in getting targeted policies to combat the spread of Mpox in Pakistan.

## 6 Conclusion

Overall, the results of the current study showed that the Pakistani populace has an adequate level of awareness, attitudes, and perception of Mpox. Our findings suggested that excellent information might lead to positive attitudes and views, which is essential for reducing the disease’s escalating effects. Careful epidemiological surveillance is required to prevent the present Mpox outbreak from spreading to other non-endemic nations. Also, there was a strong correlation between participants’ knowledge and their education, gender, age, marital status, place of residence, administrative units, education, and employment status. The results of our study highlight the need to provide more excellent information about Mpox and its preventative strategies. Public awareness campaigns must be launched to increase Mpox’s adherence to preventive advice. They could also highlight the risks to public health posed by this zoonotic illness. Our research may aid in gaining knowledge of the Mpox vaccine and vaccination status among experts, laypeople, and policymakers. To combat this disease, patients must be immunized as soon as possible. Using successful strategies that promote awareness of and combat the spread of Mpox implies the active involvement of global organizations, national health authorities, governments, medical professionals, and the media sectors.

## Data Availability

The original contributions presented in the study are included in the article/**Supplementary Material**. Further inquiries can be directed to the corresponding authors.
