# Supplementary material for: Knowledge, attitude, and perception of Pakistani populations toward monkeypox: a cross-section study
Source: Front Cell Infect Microbiol. 2025 Feb 4;14:1449096. doi: 10.3389/fcimb.2024.1449096 (PMC11832577; doi:10.3389/fcimb.2024.1449096)
Supplement: Supplementary file 1 [file DataSheet1.pdf]

Knowledge, Attitude, and Perception of Pakistani population towards Monkeypox in Pakistan: A cross-sectional study

Informed consent

Hello everyone, we have conducted a survey titled **“Knowledge, attitude, and Perception of Pakistani population towards Monkeypox in Pakistan: A cross-sectional study.”** The study results will represent the Knowledge, attitudes, and Perception toward Monkeypox in Pakistani Populations. The survey may take between 3-5 minutes to complete. You will not be asked for any personal information in the survey, and your personal information will be kept strictly confidential. You have the right to participate or deny; you can withdraw from replying during your participation. The study will not benefit you financially or in any other way, but policymakers may consider the findings and lead to implementing Monkeypox treatments in Pakistan. Participants under the age of 18 should not take part in the survey.

Section 1: Socio-demographic information

1. Gender	○ Male<br>○ Female
2. Age	○ 18–30<br>○ 31–49<br>○ ≥ 50
3. Marital status	○ Single<br>○ Married
4. Residence	○ Urban<br>○ Rural
5. Administrative units	○ Khyber Pakhtunkhwa<br>○ Punjab<br>○ Balochistan<br>○ Sindh<br>○ Azad Jammu and Kashmir<br>○ Gilgit-Baltistan<br>○ Islamabad Capital Territory

6. Education	○ Read and write/Primary School<br>○ High School<br>○ Intermediate/College<br>○ Graduate<br>○ Postgraduate
7. Employment Status	○ Retired<br>○ Full-Time<br>○ Part-Time<br>○ Unemployed<br>○ Worker<br>○ Housewife

Section 2: Knowledge

1. Is monkeypox prevalent in African countries?	○ Yes<br>○ No<br>○ Don't know
2. There are many cases of monkeypox in Pakistan.	○ Yes<br>○ No<br>○ Don't know
3. Is monkeypox a viral disease infection?	○ Yes<br>○ No<br>○ Don't know
4. Does Monkeypox spread through contaminated surfaces?	○ Yes<br>○ No<br>○ Don't know
5. Monkeypox is easily transmitted from human to human	○ Yes<br>○ No<br>○ Don't know
6. Monkeypox spreads by airborne transmission	○ Yes<br>○ No<br>○ Don't know
7. Monkeypox spreads by a Bit of an infected Monkey	○ Yes<br>○ No<br>○ Don't know
8. Measures to prevent the spread of the Monkeypox	○ Yes

disease include [Proper hand washing]	○ No<br>○ Don’t know
9. Measures to prevent the spread of the Monkeypox disease include [adequate social distance from a symptomatic individual]	○ Yes<br>○ No<br>○ Don't know
10. Measures to prevent the spread of the disease include [Avoiding handshakes and hugs]	○ Yes<br>○ No<br>○ Don't know
11. Measures to prevent the spread of the disease include [Clean and disinfecting surfaces]	○ Yes<br>○ No<br>○ Don't know
12. Measures to prevent the spread of the disease include [Self-quarantine or isolation if sick]	○ Yes<br>○ No<br>○ Don't know
13. Common symptoms include [Headache]	○ Yes<br>○ No<br>○ Don't know
14. Common symptoms include [Back pain]	○ Yes<br>○ No<br>○ Don't know
15. Common symptoms include [fever]	○ Yes<br>○ No<br>○ Don't know
16. Common symptoms include [Lymphadenopathy (swollen lymph nodes)]	○ Yes<br>○ No<br>○ Don't know
17. Do monkeypox, smallpox, and chickenpox have similar signs and symptoms?	○ Yes<br>○ No<br>○ Don't know
18. Rashes on the skin are one of the signs or symptoms of human Monkeypox	○ Yes<br>○ No<br>○ Don't know
19. Papules on the skin are one of the signs or symptoms of human Monkeypox	○ Yes<br>○ No<br>○ Don't know
20. Vesicles on the skin are one of the signs or symptoms of human Monkeypox	○ Yes<br>○ No<br>○ Don't know
21. Are antivirals required in the treatment of human	○ Yes

monkeypox patients?	○ No<br>○ Don't know
22. Are people who get the chickenpox/smallpox vaccines immunized against monkeypox?	○ Yes<br>○ No<br>○ Don't know
23. Is there a specific treatment for monkeypox?	○ Yes<br>○ No<br>○ Don't know

Section 3: Attitudes

1. Attitudes [Do you agree that Monkeypox will finally be successfully controlled?]	○ Yes<br>○ No<br>○ Don't know
2. Attitudes [People must take more care of each other now]	○ Yes<br>○ No<br>○ Don't know
3. Attitudes [I will do everything I can to protect myself and my family]	○ Yes<br>○ No<br>○ Don't know
4. I think that there are currently enough prevention and control measures for monkeypox	○ Yes<br>○ No<br>○ Don't know
5. I have bad feelings toward the monkeypox virus that it might become a worldwide pandemic	○ Yes<br>○ No<br>○ Don't know
6. I think monkeypox can be transmitted to Pakistan	○ Yes<br>○ No<br>○ Don't know
7.I think that it is dangerous to travel to the country's epidemic with monkeypox	○ Yes<br>○ No<br>○ Don't know
8.I think that monkeypox can add a new burden on the healthcare system of the affected countries	○ Yes<br>○ No

	○ Don't know
9.I am interested in learning more about monkeypox	○ Yes<br>○ No<br>○ Don’t know

Section 4: Perception

1. Perceptions toward Monkeypox [During the outbreak, eating well-cooked and safely handled meat can prevent Monkeyox]	○ Yes<br>○ No<br>○ Don't know
2. Perceptions toward Monkeyox [Sick patients should share their recent travel history with health care providers]	○ Yes<br>○ No<br>○ Don't know
3. Perceptions toward Monkeyox [I discussed Monkeyox prevention with my family and friends].	○ Yes<br>○ No<br>○ Don't know
4.During interaction with the Monkeypox patient, I will wear the necessary personal protective equipment such as masks, gloves, gown, etc	○ Yes<br>○ No<br>○ Don't know
5.I will perform hand hygiene after touching the patients’ surroundings like beds, tables, doors, etc	○ Yes<br>○ No<br>○ Don't know
6. I will avoid unnecessary close contact and practice social distancing from patients and other healthcare workers	○ Yes<br>○ No<br>○ Don't know
