# Supplementary material for: Knowledge, attitude, and perception of Pakistani populations toward monkeypox: a cross-section study
Source: Front Cell Infect Microbiol. 2025 Feb 4;14:1449096. doi: 10.3389/fcimb.2024.1449096 (PMC11832577; doi:10.3389/fcimb.2024.1449096)
Supplement: Supplementary file 2 [file Table1.doc]

**Regression Analysis**

**Multiple logistic regression analysis of the participants knowledge of the MPXV**

| **Parameter Estimates** | | | | | | | | | |
| --- | --- | --- | --- | --- | --- | --- | --- | --- | --- |
| Knowledge^a^ | | B | Std. Error | Wald | df | Sig. | Exp(B) | 95% Confidence Interval for Exp(B) | |
|  |  |  |  |  |  |  |  | Lower Bound | Upper Bound |
| 0 | Intercept | -.898 | .621 | 2.091 | 1 | .148 |  |  |  |
|  | Gender  Female | 1.012 | .194 | 27.240 | 1 | .000 | 2.752 | 1.882 | 4.025 |
|  | Male | 0^b^ | . | . | 0 | . | . | . | . |
|  | Age  18-30 | -.897 | .177 | 25.590 | 1 | .000 | .408 | .288 | .577 |
|  | 31-49 | -.833 | .188 | 19.650 | 1 | .000 | .435 | .301 | .628 |
|  | ≥ 50 | 0^b^ | . | . | 0 | . | . | . | . |
|  | Marital status  Married | -2.022 | .168 | 145.603 | 1 | .000 | .132 | .095 | .184 |
|  | Single | 0^b^ | . | . | 0 | . | . | . | . |
|  | Residence  Rural | -2.434 | .173 | 196.748 | 1 | .000 | .088 | .062 | .123 |
|  | Urban | 0^b^ | . | . | 0 | . | . | . | . |
|  | Administrative units  Azad Kashmir | -1.456 | .296 | 24.163 | 1 | .000 | .233 | .131 | .417 |
|  | Balochistan | -3.052 | .402 | 57.652 | 1 | .000 | .047 | .021 | .104 |
|  | Gilgit-Baltistan | -3.947 | .350 | 127.470 | 1 | .000 | .019 | .010 | .038 |
|  | Islamabad Capital Territory | -4.527 | .612 | 54.737 | 1 | .000 | .011 | .003 | .036 |
|  | Khyber Pakhtunkhwa | -2.903 | .216 | 179.998 | 1 | .000 | .055 | .036 | .084 |
|  | Punjab | -3.675 | .304 | 145.830 | 1 | .000 | .025 | .014 | .046 |
|  | Sindh | 0^b^ | . | . | 0 | . | . | . | . |
|  | Education  Intermediate/College | -2.347 | .641 | 13.387 | 1 | .000 | .096 | .027 | .336 |
|  | Graduate | .567 | .332 | 2.911 | 1 | .088 | 1.763 | .919 | 3.383 |
|  | High School | 3.425 | .424 | 65.328 | 1 | .000 | 30.717 | 13.388 | 70.479 |
|  | Postgraduate | 3.406 | .405 | 70.864 | 1 | .000 | 30.138 | 13.637 | 66.602 |
|  | Read and write | 0^b^ | . | . | 0 | . | . | . | . |
|  | Employment Status  Full-Time | 2.283 | .553 | 17.027 | 1 | .000 | 9.806 | 3.316 | 29.002 |
|  | Housewife | -2.200 | .834 | 6.953 | 1 | .008 | .111 | .022 | .568 |
|  | Part-Time | -.287 | .595 | .233 | 1 | .629 | .750 | .234 | 2.409 |
|  | Retired | 3.085 | .557 | 30.694 | 1 | .000 | 21.860 | 7.340 | 65.098 |
|  | Unemployed | 2.559 | .555 | 21.243 | 1 | .000 | 12.919 | 4.352 | 38.351 |
|  | Worker | 0^b^ | . | . | 0 | . | . | . | . |
| a. The reference category is: 1. | | | | | | | | | |
| b. This parameter is set to zero because it is redundant. | | | | | | | | | |

**Multiple logistic regression analysis of the participants attitude of the MPXV**

| **Parameter Estimates** | | | | | | | | | |
| --- | --- | --- | --- | --- | --- | --- | --- | --- | --- |
| Attitude^a^ | | B | Std. Error | Wald | df | Sig. | Exp(B) | 95% Confidence Interval for Exp(B) | |
|  |  |  |  |  |  |  |  | Lower Bound | Upper Bound |
| 0 | Intercept | -20.427 | 1.010 | 409.019 | 1 | .000 |  |  |  |
|  | Gender  Female | -.215 | .305 | .497 | 1 | .481 | .807 | .444 | 1.466 |
|  | Male | 0^b^ | . | . | 0 | . | . | . | . |
|  | Age  18-30 | .665 | .279 | 5.671 | 1 | .017 | 1.944 | 1.125 | 3.359 |
|  | 31-49 | -.163 | .287 | .322 | 1 | .571 | .850 | .485 | 1.490 |
|  | ≥ 50 | 0^b^ | . | . | 0 | . | . | . | . |
|  | Marital status  Married | -.776 | .197 | 15.531 | 1 | .000 | .460 | .313 | .677 |
|  | Single | 0^b^ | . | . | 0 | . | . | . | . |
|  | Residence  Rural | .737 | .192 | 14.750 | 1 | .000 | 2.090 | 1.435 | 3.046 |
|  | Urban | 0^b^ | . | . | 0 | . | . | . | . |
|  | Administrative units  Azad Kashmir | .330 | .573 | .333 | 1 | .564 | 1.392 | .453 | 4.276 |
|  | Balochistan | 1.678 | .629 | 7.108 | 1 | .008 | 5.355 | 1.560 | 18.389 |
|  | Gilgit-Baltistan | 1.448 | .535 | 7.318 | 1 | .007 | 4.253 | 1.490 | 12.138 |
|  | Islamabad Capital Territory | 1.282 | .988 | 1.685 | 1 | .194 | 3.605 | .520 | 25.001 |
|  | Khyber Pakhtunkhwa | 1.462 | .429 | 11.600 | 1 | .001 | 4.315 | 1.860 | 10.009 |
|  | Punjab | 1.603 | .482 | 11.071 | 1 | .001 | 4.967 | 1.932 | 12.768 |
|  | Sindh | 0^b^ | . | . | 0 | . | . | . | . |
|  | Intermediate/College | 15.074 | .490 | 945.427 | 1 | .000 | 3519281.164 | 1346361.934 | 9199116.225 |
|  | Graduate | 13.940 | .495 | 792.121 | 1 | .000 | 1132529.429 | 428994.512 | 2989835.234 |
|  | High School | 17.081 | .186 | 8413.259 | 1 | .000 | 26191600.081 | 18182348.739 | 37728894.362 |
|  | Postgraduate | 17.112 | .000 | . | 1 | . | 27026919.484 | 27026919.484 | 27026919.484 |
|  | Read and write | 0^b^ | . | . | 0 | . | . | . | . |
|  | Employment Status  Full-Time | .619 | .852 | .527 | 1 | .468 | 1.857 | .349 | 9.873 |
|  | Housewife | -16.988 | 1443.398 | .000 | 1 | .991 | 4.191E-008 | .000 | .^c^ |
|  | Part-Time | -2.426 | .921 | 6.932 | 1 | .008 | .088 | .015 | .538 |
|  | Retired | .304 | .880 | .119 | 1 | .730 | 1.355 | .242 | 7.600 |
|  | Unemployed | .057 | .867 | .004 | 1 | .947 | 1.059 | .194 | 5.789 |
|  | Worker | 0^b^ | . | . | 0 | . | . | . | . |
| a. The reference category is: 1. | | | | | | | | | |
| b. This parameter is set to zero because it is redundant. | | | | | | | | | |
| c. Floating point overflow occurred while computing this statistic. Its value is therefore set to system missing. | | | | | | | | | |

**Multiple logistic regression analysis of the participants attitude of the MPXV**

| **Parameter Estimates** | | | | | | | | | |
| --- | --- | --- | --- | --- | --- | --- | --- | --- | --- |
| Attitude^a^ | | B | Std. Error | Wald | df | Sig. | Exp(B) | 95% Confidence Interval for Exp(B) | |
|  |  |  |  |  |  |  |  | Lower Bound | Upper Bound |
| 0 | Intercept | -20.427 | 1.010 | 409.019 | 1 | .000 |  |  |  |
|  | Gender  Female | -.215 | .305 | .497 | 1 | .481 | .807 | .444 | 1.466 |
|  | Male | 0^b^ | . | . | 0 | . | . | . | . |
|  | Age  18-30 | .665 | .279 | 5.671 | 1 | .017 | 1.944 | 1.125 | 3.359 |
|  | 31-49 | -.163 | .287 | .322 | 1 | .571 | .850 | .485 | 1.490 |
|  | ≥50 | 0^b^ | . | . | 0 | . | . | . | . |
|  | Marital status  Married | -.776 | .197 | 15.531 | 1 | .000 | .460 | .313 | .677 |
|  | Single | 0^b^ | . | . | 0 | . | . | . | . |
|  | Residence  Rural | .737 | .192 | 14.750 | 1 | .000 | 2.090 | 1.435 | 3.046 |
|  | Urban | 0^b^ | . | . | 0 | . | . | . | . |
|  | Administrative units  Azad Kashmir | .330 | .573 | .333 | 1 | .564 | 1.392 | .453 | 4.276 |
|  | Balochistan | 1.678 | .629 | 7.108 | 1 | .008 | 5.355 | 1.560 | 18.389 |
|  | Gilgit-Baltistan | 1.448 | .535 | 7.318 | 1 | .007 | 4.253 | 1.490 | 12.138 |
|  | Islamabad Capital Territory | 1.282 | .988 | 1.685 | 1 | .194 | 3.605 | .520 | 25.001 |
|  | Khyber Pakhtunkhwa | 1.462 | .429 | 11.600 | 1 | .001 | 4.315 | 1.860 | 10.009 |
|  | Punjab | 1.603 | .482 | 11.071 | 1 | .001 | 4.967 | 1.932 | 12.768 |
|  | Sindh | 0^b^ | . | . | 0 | . | . | . | . |
|  | Intermediate/College | 15.074 | .490 | 945.427 | 1 | .000 | 3519281.164 | 1346361.934 | 9199116.225 |
|  | Graduate | 13.940 | .495 | 792.121 | 1 | .000 | 1132529.429 | 428994.512 | 2989835.234 |
|  | High School | 17.081 | .186 | 8413.259 | 1 | .000 | 26191600.081 | 18182348.739 | 37728894.362 |
|  | Postgraduate | 17.112 | .000 | . | 1 | . | 27026919.484 | 27026919.484 | 27026919.484 |
|  | Read and write | 0^b^ | . | . | 0 | . | . | . | . |
|  | Employment Status  Full-Time | .619 | .852 | .527 | 1 | .468 | 1.857 | .349 | 9.873 |
|  | Housewife | -16.988 | 1443.398 | .000 | 1 | .991 | 4.191E-008 | .000 | .^c^ |
|  | Part-Time | -2.426 | .921 | 6.932 | 1 | .008 | .088 | .015 | .538 |
|  | Retired | .304 | .880 | .119 | 1 | .730 | 1.355 | .242 | 7.600 |
|  | Unemployed | .057 | .867 | .004 | 1 | .947 | 1.059 | .194 | 5.789 |
|  | Worker | 0^b^ | . | . | 0 | . | . | . | . |
| a. The reference category is: 1. | | | | | | | | | |
| b. This parameter is set to zero because it is redundant. | | | | | | | | | |
| c. Floating point overflow occurred while computing this statistic. Its value is therefore set to system missing. | | | | | | | | | |
